# Reprogramming Mitochondrial Adaptation: LONP1 at the Crossroads of Proteostasis, Metabolism, and Disease

**DOI:** 10.3390/antiox15070793

**Published:** 2026-06-25

**Authors:** Hsu-Hung Chang, Phebe Ting Syuan Chang, Chung-Che Tsai, Chan-Yen Kuo

**Affiliations:** 1 Division of Nephrology, Department of Internal Medicine, Sijhih Cathay General Hospital, New Taipei City 221, Taiwan; hhchang@cgh.org.tw; 2 School of Biological Sciences, University of California, San Diego, CA 92093, USA; p6chang@ucsd.edu; 3 Department of Research, Taipei Tzu Chi Hospital, The Buddhist Tzu Chi Medical Foundation, New Taipei City 231, Taiwan; 114009@ctcn.edu.tw; 4 Institute of Oral Medicine and Materials, College of Medicine, Tzu Chi University, Hualien 970, Taiwan

**Keywords:** LONP1, mitochondrial proteostasis, mitochondrial metabolism, stress response, cancer metabolism

## Abstract

Mitochondrial Lon peptidase 1 (LONP1) is an ATP-dependent AAA^+^ (ATPases associated with diverse cellular activities) protease that has emerged as a key regulator of mitochondrial proteostasis, with functions extending beyond protein quality control. In addition to degrading misfolded and oxidized proteins, LONP1 coordinates mitochondrial DNA maintenance, metabolic remodeling, and stress-responsive signaling. Recent structural and functional advances have expanded the biological significance of LONP1 beyond protein quality control, highlighting its roles in mitochondrial metabolism, genome maintenance, and stress responses. LONP1 dysregulation is increasingly implicated in cancer, metabolic disorders, neurodegeneration, and aging, where it exerts context-dependent effects on cell survival and disease progression. In cancer, LONP1 supports metabolic plasticity, redox adaptation, and therapeutic resistance, whereas in degenerative conditions, its decline contributes to mitochondrial dysfunction and tissue damage. Here, we synthesize recent insights into the structure, mechanisms, and biological functions of LONP1 and discuss their implications for human disease. We further discuss emerging therapeutic strategies and key challenges for targeting LONP1 in human disease.

## 1. Introduction

Mitochondria are multifunctional organelles that contribute to cellular energy production, metabolic regulation, and apoptosis. The maintenance of mitochondrial function requires a tightly regulated protein quality control (PQC) system to ensure the proper folding, assembly, and turnover of mitochondrial proteins. Among the key components of this system, mitochondrial Lon peptidase 1 (LONP1) has emerged as a critical regulator of mitochondrial proteostasis [1,2,3]. LONP1 is a nuclear-encoded, mitochondrial matrix-localized AAA^+^ (ATPases associated with diverse cellular activities) protease that forms homohexameric complexes and utilizes ATP hydrolysis to drive substrate unfolding and degradation [4,5,6]. Initially characterized as a protease targeting misfolded proteins, LONP1 is now recognized as a multifunctional enzyme that integrates proteolysis, chaperone activity, and mitochondrial DNA (mtDNA) regulation [7]. Recent studies have expanded the functional landscape of LONP1 beyond protein degradation, revealing important roles in mitochondrial genome maintenance, metabolic regulation, and cellular stress adaptation [5,8,9].

Although extensive studies have been conducted on mitochondrial proteases, no unifying framework has been established, explaining how LONP1 coordinates diverse mitochondrial functions across physiological and pathological conditions [10]. Therefore, this review aims not only to summarize recent advances but also conceptualize LONP1 as a dynamic regulatory hub that links mitochondrial function to cellular adaptation and disease progression.

## 2. Structural and Biochemical Features of LONP1

### 2.1. Domain Organization

LONP1 comprises three conserved functional domains: an N-terminal domain responsible for substrate recognition and binding, a central AAA^+^ ATPase domain that mediates ATP binding and hydrolysis to drive conformational changes, and a C-terminal protease domain that catalyzes substrate degradation. Structurally, LONP1 forms a homohexameric ring complex with a central axial pore through which substrate proteins are unfolded and translocated into the proteolytic chamber for ATP-dependent degradation, as revealed in recent cryo-electron microscopy and biochemical studies [4,11,12].

### 2.2. ATP-Dependent Proteolytic Mechanism

LONP1 maintains mitochondrial proteostasis through a highly coordinated, ATP-dependent proteolytic cycle in which misfolded or oxidatively damaged substrates are initially recognized by the N-terminal domain. Thereafter, ATP binding and hydrolysis occurs within the AAA^+^ module, which drives conformational remodeling to mechanically unfold client proteins and translocate them through the central pore into the proteolytic chamber for degradation [11,13]. Recent cryo-electron microscopy and biochemical studies further demonstrated that substrate engagement is coupled to a dynamic, stepwise translocation mechanism. This mechanism is mediated by conserved pore loops arranged in a spiral staircase configuration, consistent with a processive “hand-over-hand” model of substrate threading, while distinct nucleotide states coordinate sequential conformational transitions across subunits to ensure directional translocation and catalytic efficiency [5,7,13]. Importantly, emerging evidence indicates that ATPase activity and proteolytic function can be partially uncoupled. This finding reveals an additional layer of allosteric regulation in which ATP hydrolysis does not strictly dictate degradation kinetics but instead modulates substrate processing in a context-dependent manner, thereby enabling LONP1 to flexibly adapt its proteolytic output to fluctuations in mitochondrial stress and metabolic demand [14].

### 2.3. Regulation of LONP1 Expression and Activity

Upstream regulators reported to influence LONP1 expression include hypoxia-associated signaling, oxidative stress-responsive pathways, mitochondrial unfolded protein response (UPRmt), and metabolic stress-associated signaling networks, although the relative contribution of each pathway appears to be tissue- and context-dependent [7,15,16,17]. These regulatory mechanisms enable cells to dynamically adjust LONP1 expression and activity in response to fluctuations in mitochondrial stress and metabolic demand [7,18].

LONP1 expressions and activity are dynamically regulated by multiple cellular stress signals and mitochondrial quality control pathways. Under conditions of oxidative stress, hypoxia, mitochondrial protein misfolding, and metabolic perturbation, LONP1 expression is frequently induced as part of an adaptive response that preserves mitochondrial proteostasis and bioenergetic function [7,18]. Mitochondrial stress activates integrated stress response pathways involving ATF4, CHOP, and ATF5, which coordinate the transcriptional program of the mammalian UPRmt and promote the expression of mitochondrial proteostasis genes [15,16,17]. In addition, hypoxia- and oxidative stress-associated signaling pathways have been implicated in the regulation of LONP1 expression and activity under specific pathological conditions [18,19]. Beyond transcriptional regulation, LONP1 activity is influenced by ATP-dependent conformational cycling that governs substrate engagement, translocation, and proteolytic activation [12]. These regulatory mechanisms enable LONP1 to coordinate mitochondrial protein quality control, metabolic adaptation, and stress responses under changing physiological and pathological conditions.

## 3. Biological Functions of LONP1 in Mitochondrial Homeostasis

### 3.1. Mitochondrial Protein Quality Control

LONP1 contributes to mitochondrial protein quality control (PQC) as an ATP-dependent matrix protease that selectively degrades misfolded, oxidatively damaged, and unassembled respiratory chain proteins, thereby maintaining mitochondrial protein homeostasis and oxidative phosphorylation (OXPHOS) capacity [4,20]. Mechanistically, LONP1 cooperates with other mitochondrial proteostasis factors to limit the accumulation of misfolded and aggregation-prone proteins, whereas its depletion or dysfunction leads to the accumulation of mitochondrial protein aggregates, impaired assembly and stability of electron transport chain (ETC) complexes, and consequent mitochondrial dysfunction and bioenergetic failure [21,22,23]. LONP1 preserves ETC integrity by facilitating the turnover of oxidatively damaged or improperly assembled respiratory chain proteins, including components involved in Complex I and Complex IV biogenesis [24,25]. Studies have shown that LONP1 participates in the quality control of respiratory chain proteins and prevents the accumulation of dysfunctional ETC components that promote electron leakage and respiratory impairment [7,24]. By maintaining the integrity of Complex I and Complex III, two major sites of mitochondrial ROS generation, LONP1 limits excessive mtROS production and preserves respiratory efficiency [7,26]. Through these quality control functions, LONP1 supports the stability and activity of electron transport chain complexes and protects against oxidative stress-induced mitochondrial dysfunction [7,25].

This phenotype highlights LONP1 as being not merely a degradative enzyme but also a critical determinant of mitochondrial proteome integrity and respiratory competence under both basal and stress conditions.

### 3.2. Chaperone-like Activity and Protein Folding

In addition to its proteolytic activity, LONP1 exerts chaperone-like functions that contribute to mitochondrial proteostasis by facilitating protein folding and limiting aggregation, particularly under proteotoxic stress. Early biochemical and structural studies demonstrated that LONP1 can bind unfolded polypeptides and, in an ATP-dependent manner, promote their refolding or stabilization independent of proteolysis [25,27]. In contrast, recent work supports a model in which LONP1 operates within the mitochondrial PQC network functionally coordinated with chaperones, such as mitochondrial heat shock protein 70 (mtHSP70) and HSP60, to manage the fate of newly imported or misfolded proteins by balancing folding versus degradation decisions [21,28,29].

### 3.3. Regulation of Mitochondrial DNA Maintenance

LONP1 functions as not only a mitochondrial protease but also a DNA-binding protein that associates with mitochondrial nucleoids and modulates the stability of mitochondrial transcription factor A (TFAM), an important regulator of mtDNA organization and gene expression [30]. By controlling TFAM degradation and maintaining the TFAM/mtDNA ratio, LONP1 finetunes mitochondrial transcriptional activity, replication efficiency, and genome stability [31]. Recent studies have further indicated that LONP1 influences mtDNA homeostasis through the TFAM–POLG axis and contributes to mitochondrial genome maintenance under stress conditions, thereby coordinating mtDNA replication, transcription, and integrity [32]. By regulating TFAM turnover and maintaining an appropriate TFAM/mtDNA ratio, LONP1 influences mtDNA replication and mitochondrial gene expression, thereby affecting respiratory chain function, cellular bioenergetics, stress adaptation, and susceptibility to apoptosis [4,33]. Through this mechanism, LONP1 affects respiratory chain activity, cellular bioenergetics, stress adaptation, and susceptibility to apoptosis.

### 3.4. Metabolic Regulation and Bioenergetic Adaptation

LONP1 is increasingly recognized as a critical regulator of mitochondrial metabolic homeostasis, integrating proteostasis with bioenergetic and metabolic control rather than functioning solely as a matrix protease [6,26]. Recent studies have demonstrated that LONP1 preserves mitochondrial respiratory chain integrity and supports oxidative metabolism by maintaining mitochondrial protein quality control (PQC), thereby sustaining electron transport chain (ETC) function and oxidative phosphorylation (OXPHOS) capacity. Genetic and developmental studies further indicate that Lonp1 is required for metabolic remodeling toward mitochondrial OXPHOS during cardiac development, whereas its deficiency results in impaired mitochondrial respiration and defective metabolic maturation [9,18,22]. In addition to its role in mitochondrial proteostasis, LONP1 contributes to the maintenance of respiratory chain function by facilitating the turnover of oxidatively damaged, misfolded, or unassembled mitochondrial proteins [24,25]. Through these quality control activities, LONP1 preserves ETC stability, limits mitochondrial ROS accumulation, and protects against oxidative stress-induced mitochondrial dysfunction [7,26]. Importantly, Quirós et al. demonstrated that LONP1 regulates mitochondrial bioenergetics and oxidative metabolism, thereby contributing to adaptive metabolic remodeling under cellular stress conditions [26]. Beyond maintaining ETC integrity, LONP1 contributes to metabolic remodeling through the regulation of specific mitochondrial metabolic proteins. Recent studies have demonstrated that LONP1 promotes the degradation of mitochondrial pyruvate carrier 1 (MPC1), thereby reducing pyruvate transport into mitochondria and favoring glycolytic metabolism in cancer cells [34]. In addition, LONP1 regulates the stability of aconitase 2 (ACO2), a key tricarboxylic acid cycle enzyme, thereby influencing mitochondrial iron metabolism, oxidative phosphorylation, and ferroptosis susceptibility [35]. LONP1 has also been implicated in the maintenance of respiratory chain proteins and oxidative metabolic capacity under conditions of mitochondrial stress [7,26]. Together, these findings indicate that LONP1-mediated metabolic remodeling is achieved through the regulation of specific mitochondrial substrates rather than through global effects on metabolism.

These observations suggest that mitochondrial proteostasis is not merely a quality control mechanism but also a determinant of metabolic adaptation. By coordinating PQC with respiratory chain maintenance and mitochondrial bioenergetics, LONP1 influences cellular energy production and metabolic homeostasis. Notably, LONP1 promotes metabolic adaptation under stress conditions through the regulation of specific mitochondrial substrates. By facilitating MPC1 degradation, LONP1 limits mitochondrial pyruvate utilization and favors glycolytic metabolism, whereas the regulation of ACO2 stability influences tricarboxylic acid cycle activity, oxidative phosphorylation, and ferroptosis susceptibility [34,35]. These substrate-specific effects enable cells to adjust mitochondrial energy metabolism in response to bioenergetic stress. Collectively, these findings demonstrate that LONP1 contributes to metabolic regulation through its effects on mitochondrial protein quality control, respiratory function, and enzyme homeostasis.

**Figure 1:**
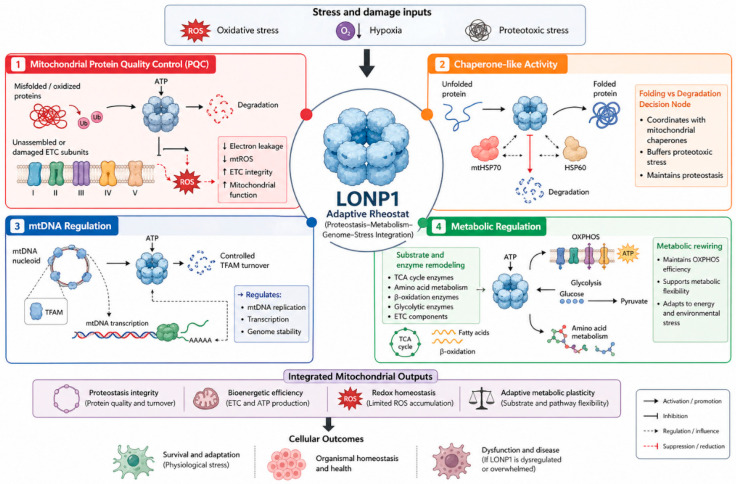
Core biological functions of mitochondrial Lon peptidase 1 (LONP1) in mitochondrial regulation. LONP1 coordinates mitochondrial protein quality control, chaperone-assisted proteostasis, mitochondrial DNA maintenance, and metabolic adaptation. Through these integrated functions, LONP1 supports mitochondrial integrity and cellular homeostasis under physiological and stress conditions. HSP, heat shock protein; mtROS, mitochondrial reactive oxygen species; OXPHOS, oxidative phosphorylation; TCA, tricarboxylic acid. This figure was generated with the assistance of ChatGPT (OpenAI, GPT-5.5) and subsequently curated by the authors to ensure accuracy in biological representation, terminology, and conceptual integration.

Importantly, these functions should not be viewed as independent processes but rather as interconnected components of a unified mitochondrial adaptive network. Through the coordinated regulation of protein quality control, mitochondrial genome maintenance, and metabolic homeostasis, LONP1 enables mitochondria to adapt to fluctuating bioenergetic and environmental demands. As summarized in Figure 1, this integrative role positions LONP1 as a central regulator of mitochondrial resilience under both physiological and stress conditions.

### 3.5. LONP1 as a Gatekeeper of Mitochondrial Protein Import

Emerging evidence indicates that LONP1 acts as a gatekeeper of mitochondrial protein import by functionally coupling with mitochondrial import and chaperone machinery, including mtHSP70-dependent systems, to surveil newly translocated polypeptides. Upon entry into the matrix, LONP1 associates with nascent precursor proteins, facilitating their proper folding through chaperone-like activity while simultaneously recognizing and selectively degrading improperly processed or misfolded substrates through its ATP-dependent protease function, thereby coordinating protein maturation and quality control at the point of import. This dual folding–degradation mechanism is essential for maintaining mitochondrial proteostasis, preventing the accumulation of toxic protein aggregates, and preserving respiratory chain integrity and organelle function [4,36]. Moreover, this gatekeeping function suggests that LONP1 acts at the earliest stage of mitochondrial proteostasis, ensuring that only properly folded and functional proteins are incorporated into the mitochondrial network, thereby preventing the propagation of proteotoxic stress.

### 3.6. Integration of Cellular Stress Responses

Rather than acting solely as a stress-responsive protease, LONP1 contributes to mitochondrial stress responses through its effects on protein quality control (PQC), redox regulation, and metabolic function, thereby helping maintain mitochondrial performance during cellular stress. By modulating mitochondrial proteome integrity alongside ROS-dependent signaling pathways, LONP1 enables a graded transition from adaptive remodeling to stress-induced dysfunction [3,5,8,18,23].

LONP1-mediated stress responses are tightly intertwined with the role of LONP1 in proteostasis, mtDNA regulation, and metabolic control, collectively forming an integrated adaptive network that allows mitochondria to dynamically respond to environmental and intracellular stress signals. Under conditions of oxidative stress, hypoxia, and proteotoxic burden, LONP1 is consistently upregulated, where it facilitates the selective removal of damaged mitochondrial proteins, preserves ETC integrity, and supports metabolic reprogramming toward stress-adaptive states [1,3,8,18,37]. In addition to maintaining redox balance, LONP1-dependent regulation of mitochondrial ROS (mtROS) functions as a signaling interface that influences multiple stress-responsive pathways, including ERK, JNK, and p38 MAPK signaling, hypoxia-inducible factor 1 alpha (HIF-1α), nuclear factor kappa B (NF-κB), and integrated stress response pathways mediated by ATF4 and CHOP. Through the modulation of mitochondrial redox status, LONP1 contributes to the coordination of cellular adaptation, inflammatory signaling, metabolic reprogramming, and cell fate decisions under stress conditions [7,24,26,38,39]. Collectively, these findings demonstrate that LONP1 plays an important role in preserving mitochondrial function during cellular stress through its effects on protein quality control, energy metabolism, and stress-responsive pathways.

## 4. LONP1 in Cancer

LONP1 has emerged as a critical mitochondrial regulator in cancer, where it supports tumor progression through the coordinated control of proteostasis, metabolism, and stress adaptation rather than through isolated pathways. Accumulating evidence indicates that LONP1 promotes cancer cell fitness and tumor progression through three major interconnected mechanisms: metabolic reprogramming, redox adaptation, and proteostasis buffering. In addition, LONP1 contributes to therapeutic resistance by enhancing mitochondrial stress tolerance and adaptive survival pathways. Moreover, LONP1 is frequently upregulated across multiple malignancies and is associated with poor clinical outcomes [19,26]. To systematically integrate the emerging roles of LONP1 in cancer, the key mechanistic axes and supporting evidence underlying its oncogenic functions are summarized in Table 1. The table highlights how LONP1 coordinates metabolic reprogramming, redox adaptation, proteostasis buffering, and therapeutic resistance, thereby linking mitochondrial quality control to tumor cell fitness.

First, LONP1 drives metabolic reprogramming by modulating mitochondrial enzyme stability and bioenergetic pathways. In prostate cancer, LONP1 promotes glycolytic reprogramming through the degradation of mitochondrial pyruvate carrier components, thereby enhancing tumor growth and metastatic potential [34]. Similarly, LONP1-dependent regulation of mitochondrial metabolism has been linked to altered substrate utilization and bioenergetic flexibility in proliferative disease contexts [3].

Second, LONP1 contributes to redox adaptation by maintaining mitochondrial respiratory chain homeostasis and modulating mitochondrial ROS (mtROS) signaling. Through the quality control of ETC components, LONP1 influences mitochondrial redox status and prevents excessive oxidative damage while permitting stress-adaptive ROS signaling. Emerging evidence suggests that LONP1-dependent mtROS regulation can influence multiple downstream pathways, including ERK/JNK/p38 MAPK, HIF-1α, and NF-κB signaling, which collectively contribute to cellular adaptation, survival, metabolic reprogramming, and, in specific cancer contexts, EMT and metastatic progression [7,24,26,48].

Third, LONP1 acts as a proteostasis buffer that enables cancer cells to tolerate mitochondrial stress. By selectively degrading misfolded or damaged proteins and coordinating with other mitochondrial proteases, such as the caseinolytic mitochondrial matrix peptidase proteolytic subunit (CLPP), LONP1 prevents the accumulation of toxic protein aggregates and preserves mitochondrial integrity [23,41]. This buffering capacity is particularly important under conditions of hypoxia, nutrient limitation, and rapid proliferation.

Beyond its tumor-promoting functions, LONP1 also contributes to adaptive cancer phenotypes, particularly therapeutic resistance. By compensating for impaired protein degradation pathways and enhancing mitochondrial stress tolerance, LONP1 promotes cancer cell survival under therapeutic challenge. In multiple myeloma, LONP1 overexpression confers resistance to proteasome inhibitors by sustaining mitochondrial proteostasis [46]. Consistently, dual-targeting strategies against LONP1 and proteasome activity have demonstrated enhanced anti-tumor effects in preclinical models [47]. In addition, LONP1 modulation influences ferroptosis sensitivity in a context-dependent manner [42]. Beyond these core mechanisms, LONP1 also regulates the EMT, autophagy, and ferroptosis across multiple cancer types, including pancreatic, hepatocellular, cervical, and colorectal cancers [35,43,44,45]. Collectively, these findings position LONP1 as a context-dependent regulator of cancer cell fitness that integrates mitochondrial proteostasis with metabolic and signaling networks. Considering all of the above, LONP1 should not be viewed merely as a mitochondrial quality control protease but rather as a non-oncogene addiction factor that cancer cells rely on to sustain mitochondrial function, metabolic flexibility, and stress resilience.

**Figure 2:**
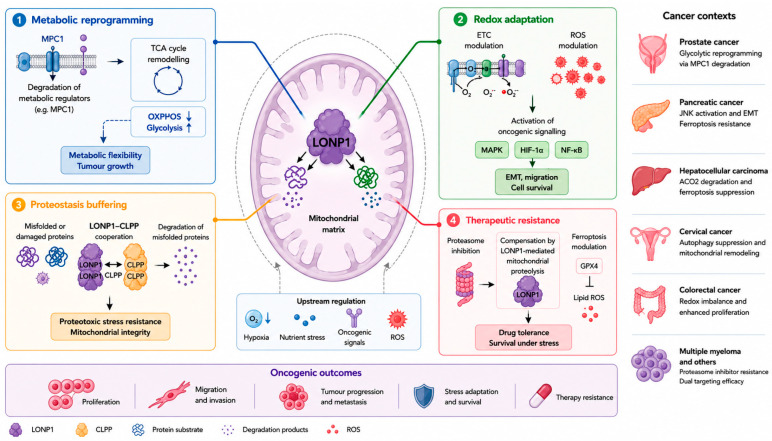
Role of LONP1 in cancer-associated mitochondrial adaptation. LONP1 promotes metabolic reprogramming, redox adaptation, proteostasis buffering, and therapeutic resistance, thereby supporting tumor cell survival, proliferation, and disease progression. ACO2, aconitase 2; GPX4, glutathione peroxidase 4; JNK, c-Jun N-terminal kinase; MPC1, mitochondrial pyruvate carrier 1. The graphical summary was initially generated using ChatGPT (OpenAI, GPT-5.5) and further edited and validated by the authors to ensure the accuracy of biological mechanisms, terminology, and visual representation. ↑ Upregulation; ↓ Downregulation.

In cancer, elevated LONP1 expression promotes mitochondrial function and cellular survival, thereby facilitating tumor growth, therapeutic resistance, and disease progression (Figure 2). Through the coordinated control of mitochondrial protein quality, bioenergetic pathways, and redox homeostasis, LONP1 promotes metabolic flexibility, supports stress tolerance, and facilitates the activation of oncogenic signaling networks. These interconnected processes collectively enhance tumor cell survival, proliferation, and therapeutic resistance, highlighting LONP1 as a key determinant of cancer cell fitness under adverse conditions.

## 5. LONP1 in Metabolic Disorders

Beyond cancer, LONP1 plays a critical role in metabolic tissues where mitochondrial function is essential for energy homeostasis [19]. LONP1 deficiency disrupts mitochondrial protein-folding homeostasis in pancreatic β-cells, resulting in mitochondrial proteotoxic stress, impaired oxidative phosphorylation (OXPHOS), and reduced ATP production [22]. Because glucose-stimulated insulin secretion is highly dependent on mitochondrial ATP generation, these defects impair insulin secretion and promote β-cell dysfunction. Persistent mitochondrial stress further activates apoptotic pathways and reduces β-cell mass, ultimately leading to glucose intolerance and hyperglycemia [22,49]. Similar findings have indicated that LONP1 deficiency induces mitochondrial proteotoxic stress in β-cells, impairing respiratory function and triggering β-cell loss. This response culminates in defective insulin secretion and glucose intolerance, highlighting LONP1 as a key regulator of β-cell survival and metabolic homeostasis [49]. In addition, LONP1 is indispensable for cardiac metabolic maturation as it maintains mitochondrial proteostasis during the developmental shift toward OXPHOS. LONP1 loss disrupts mitochondrial PQC, leading to impaired respiratory chain function, defective oxidative metabolism, and metabolic reprogramming failure, ultimately resulting in compromised cardiac function [18]. Muthu et al. reported that cardiomyocyte-specific *Lonp1* haploinsufficiency induces early mitochondrial stress, characterized by the impaired expression of OXPHOS genes, activation of mitochondrial stress responses, and mild cardiac dysfunction. In contrast, systemic *Lonp1* haploinsufficiency preserves cardiac function, likely through compensatory inter-organ signaling that mitigates mitochondrial stress [50]. In addition, LONP1 protects the heart from ischemic injury by maintaining mitochondrial redox balance and suppressing oxidative damage-induced apoptosis [51].

LONP1 loss drives metabolic dysfunction-associated steatohepatitis-associated fibrosis by disrupting dihydroorotate dehydrogenase (DHODH)-dependent metabolic control and promoting orotic acid accumulation. This metabolite-dependent signaling activates hepatic stellate cells by activating transcription factor 3 (ATF3), identifying the LONP1–DHODH–orotic acid axis as a tractable target for anti-fibrotic intervention [52]. Contrastingly, skeletal muscle LONP1 deficiency elicits mitochondrial stress that activates systemic adaptive signaling, leading to improved metabolic resilience. This exemplifies mitohormesis, where mitochondrial proteostasis imbalance drives organism-wide metabolic reprogramming [53]. LONP1 is involved in both mitochondrial and endoplasmic reticulum (ER) stress responses through its association with UPRmt and UPRER signaling pathways. In high-energy tissues such as the heart, LONP1 supports mitochondrial function and protein quality control, whereas its deficiency results in mitochondrial dysfunction and impaired cardiac performance [54].

**Figure 3:**
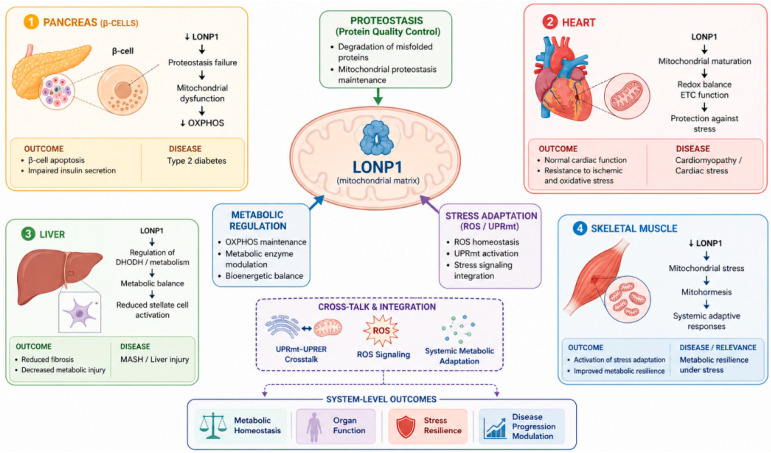
LONP1 coordinates mitochondrial adaptation across metabolic tissues. Through its roles in mitochondrial proteostasis, metabolism, and stress adaptation, LONP1 influences β-cell function, cardiac homeostasis, hepatic metabolism, and skeletal muscle responses. DHODH, dihydroorotate dehydrogenase; UPRmt, mitochondrial unfolded protein response. The graphical summary was generated with the assistance of ChatGPT (OpenAI, GPT-5.5) and subsequently refined and validated by the authors to ensure the accuracy of biological mechanisms, terminology, and visual representation. ↓ Downregulation.

Collectively, these findings highlight the diverse roles of LONP1 in metabolic and systemic disorders. As summarized in Table 2 and Figure 3, LONP1 influences mitochondrial function, metabolic homeostasis, and tissue adaptation across multiple organs, thereby contributing to disease susceptibility and progression [23,35,43,55].

## 6. LONP1 in Aging and Degenerative Diseases

LONP1 has emerged as a key determinant of mitochondrial resilience in aging and degenerative diseases, particularly in tissues that are highly dependent on mitochondrial homeostasis and stress adaptation, including the brain, skeletal muscle, kidney, ovary, and lung [25,56,57,58,59,60,61]. Rather than representing a passive consequence of aging, reduced or dysregulated LONP1 activity may actively contribute to age-associated mitochondrial dysfunction by impairing proteostasis, respiratory efficiency, and stress response capacity [25,62]. Rather than a passive consequence of aging, reduced or dysregulated LONP1 activity may actively drive age-associated mitochondrial failure by weakening proteostasis buffering capacity, impairing respiratory efficiency, and amplifying oxidative stress [25,62]. In neurons, LONP1 preserves mitochondrial proteome integrity and limits oxidative injury; its dysfunction promotes proteotoxic stress, bioenergetic collapse, and neuronal vulnerability, thereby contributing to neurodegenerative phenotypes [56]. Consistently, the ATF3–LONP1 axis protects against cerebral ischemia–reperfusion injury by maintaining mitochondrial integrity and suppressing oxidative stress-induced neuronal apoptosis [63], whereas pathogenic LONP1 mutations impair mitochondrial enzyme turnover, disrupt pyruvate dehydrogenase activity, and cause severe neurodegeneration with progressive cerebellar atrophy [64].

Aging-related decline in LONP1 further links mitochondrial quality control failure to tissue degeneration. In osteoarthritis, reduced LONP1 expression in aged chondrocytes promotes mitochondrial dysfunction, oxidative stress, defective mitophagy, and MAPK activation, thereby accelerating cartilage degeneration [57]. In the hippocampus, aging disrupts LONP1 regulation through a *Mecp2*-associated epigenetic mechanism, producing a mismatch between *Lonp1* transcription and protein abundance that may compromise mitochondrial proteostasis and cognitive function [65]. Experimental inhibition of LONP1 similarly disrupts hippocampal energy metabolism and redox balance, leading to synaptic dysfunction and aging-like cognitive impairment [66]. Sex-specific differences have also been reported, with aged male mice showing more pronounced reductions in LONP1-dependent mitochondrial proteostasis and increased oxidative stress, suggesting that LONP1 decline may contribute to differential patterns of brain aging [67].

Beyond the nervous system, LONP1 insufficiency contributes to degeneration across multiple organs. In oocytes, LONP1 maintains mitochondrial integrity and prevents AIFM1-mediated apoptosis, thereby supporting follicular development and fertility [58]. In skeletal muscle, LONP1 loss impairs mitochondrial respiration, promotes the accumulation of abnormal mitochondrial proteins, activates autophagy–lysosome pathways, and contributes to muscle wasting and reduced exercise capacity [59]. In aged kidneys, reduced LONP1 expression is associated with mitochondrial damage and renal fibrosis, partly through disruption of the methyltransferase-like 3–m6A–insulin-like growth factor 2 mRNA binding protein 2 regulatory axis [60]. In the lung, *Lonp1* deficiency in alveolar type II cells promotes epithelial senescence and exacerbates pulmonary fibrosis through p53/p21 activation and FGF2-associated pro-senescent signaling [61]. Moreover, proteomic analyses indicate that LONP1 deficiency may have limited effects under basal conditions but significantly compromises mitochondrial PQC under stress, leading to increased protein aggregation and heightened vulnerability to aging-associated stressors [68].

**Figure 4:**
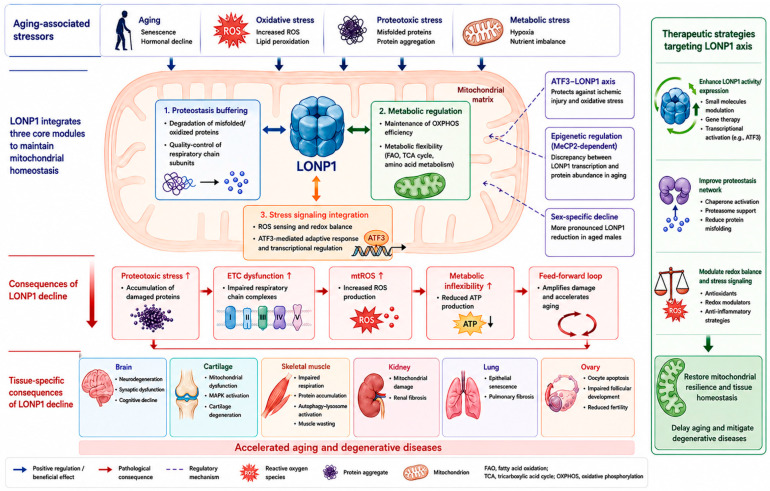
LONP1 in aging-associated mitochondrial dysfunction and tissue degeneration. Aging-related decline in LONP1 impairs mitochondrial proteostasis, respiratory function, and stress adaptation, thereby contributing to neurodegeneration, musculoskeletal decline, fibrosis, and other age-associated disorders. ATF3, activating transcription factor 3. The graphical summary was generated with the assistance of ChatGPT (OpenAI, GPT-5.5) and subsequently reviewed and manually refined by the authors to ensure the accuracy of biological mechanisms, correct use of terminology, and accurate visual representation. ↑ Upregulation; ↓ Downregulation.

Collectively, these findings support a unifying model in which LONP1 decline represents a progressive failure of mitochondrial proteostasis’ buffering capacity. This failure permits oxidized and misfolded protein accumulation, respiratory chain instability, mtROS amplification, and metabolic inflexibility, ultimately driving the activation of maladaptive stress pathways and tissue degeneration. From a systems perspective, LONP1 contributes to mitochondrial resilience by supporting protein quality control, metabolic function, and cellular responses to stress. Therefore, therapeutic strategies aimed at preserving or restoring LONP1 activity may provide a broadly applicable approach for mitigating aging-related neurodegeneration, musculoskeletal decline, renal fibrosis, reproductive aging, and pulmonary fibrosis. As illustrated in Figure 4, LONP1 influences diverse mitochondrial processes, including protein quality control, energy metabolism, mitochondrial genome maintenance, and stress response pathways. Aging-associated stress progressively impairs LONP1 activity, leading to the loss of proteostasis buffering, accumulation of damaged proteins, ETC dysfunction, and increased mtROS generation. These changes establish a feed-forward cycle of proteotoxic and redox stress that drives metabolic inflexibility, cellular dysfunction, and tissue degeneration, highlighting LONP1 as a key determinant of mitochondrial resilience during aging.

## 7. Comparative Roles of LONP1 Across Human Diseases

Although LONP1 is broadly implicated in diverse pathological conditions, its biological significance is highly context dependent. Although LONP1 is broadly implicated in diverse pathological conditions, its biological significance is highly context dependent. The consequences of altered LONP1 activity vary substantially among disease states, tissues, and metabolic environments [26,50,54]. However, the direction of its pathological effects differs substantially between disease settings. In cancer, elevated LONP1 expression frequently promotes cellular fitness, metabolic plasticity, and therapeutic resistance through the maintenance of mitochondrial proteostasis and stress adaptation [4,26,40,69]. In contrast, in aging-related and degenerative disorders, loss or decline of LONP1 function contributes to mitochondrial dysfunction, oxidative stress, impaired bioenergetics, and tissue injury [22,45,70]. These observations suggest that LONP1 should not be regarded simply as either a pathogenic factor or a protective protein but rather as a context-dependent determinant of mitochondrial resilience, whose biological consequences depend on tissue type, metabolic demand, and cellular stress burden [26,50,54].

### 7.1. LONP1 in Cancer: A Non-Oncogene Addiction Factor

In cancer, LONP1 is commonly upregulated and functions as a mitochondrial stress adaptation factor that supports tumor progression. Unlike classical oncogenes, LONP1 does not directly initiate malignant transformation but instead enables cancer cells to tolerate proteotoxic, oxidative, and metabolic stress [37,40,41]. Through its regulation of mitochondrial proteostasis, redox homeostasis, and metabolic flexibility, LONP1 enhances tumor cell survival under conditions of hypoxia, nutrient deprivation, and therapeutic challenge [26,34,46]. Emerging evidence further indicates that LONP1 contributes to metabolic reprogramming, therapy resistance, ferroptosis regulation, and cancer cell survival across multiple malignancies [34,35,43].

Notably, LONP1 promotes metabolic rewiring toward glycolytic and stress-adaptive states while simultaneously buffering mitochondrial dysfunction through selective degradation of damaged proteins. These functions contribute to therapy resistance, metastatic potential, epithelial–mesenchymal transition, and ferroptosis regulation across multiple malignancies [26,34,35,42,43,46]. Consequently, LONP1 inhibition may selectively impair mitochondrial adaptation in cancer cells and represents a promising anti-cancer strategy [8,23].

### 7.2. Protective Functions of LONP1 in Metabolic Tissues

In contrast to in cancer, LONP1 generally exerts protective effects in metabolic tissues. Pancreatic β-cells, cardiomyocytes, hepatocytes, and skeletal muscle cells rely heavily on mitochondrial function to maintain metabolic homeostasis [18,22] Loss of LONP1 disrupts mitochondrial protein quality control, impairs oxidative phosphorylation, and increases oxidative stress, ultimately resulting in cellular dysfunction and tissue injury [50,54].

Importantly, studies on type 2 diabetes, cardiac disease, and metabolic dysfunction-associated steatohepatitis indicate that LONP1 deficiency frequently precedes overt tissue damage, suggesting that mitochondrial proteostasis failure may represent an early pathogenic event [4,50,62]. Therefore, therapeutic restoration of LONP1 activity may offer opportunities to preserve mitochondrial integrity and delay disease progression.

### 7.3. LONP1 in Aging and Neurodegeneration

Aging-associated decline in LONP1 activity is increasingly recognized as a contributor to mitochondrial dysfunction and age-related tissue degeneration [25,62]. Unlike cancer, in which elevated LONP1 promotes cellular fitness, aging tissues frequently exhibit insufficient LONP1-mediated proteostasis buffering. Reduced LONP1 activity permits the accumulation of oxidized and misfolded proteins, respiratory chain instability, excessive mitochondrial ROS production, and impaired metabolic flexibility [56,57,68]. These alterations have been implicated in neurodegeneration, osteoarthritis, sarcopenia, and other aging-associated disorders [57,59,64].

This progressive decline has been implicated in neurodegenerative disorders, cognitive impairment, osteoarthritis, sarcopenia, and reproductive aging [56,57,58,59]. These observations support a model in which LONP1 functions as a mitochondrial resilience factor whose gradual loss contributes to age-related tissue degeneration. Consequently, strategies aimed at preserving or restoring LONP1 activity may represent a novel approach for healthy aging and neuroprotection [7,25].

### 7.4. Organ Injury and Fibrosis

Recent evidence suggests that LONP1 also plays an important role in tissue injury and fibrotic remodeling. In the kidney, liver, and lung, reduced LONP1 expression has been associated with impaired mitochondrial quality control, enhanced oxidative stress, and activation of tissue injury or fibrotic pathways [60,61,71,72]. Similarly, in the cardiovascular system, LONP1 contributes to mitochondrial homeostasis and protects against ischemic injury, highlighting its broader role in maintaining tissue resilience under stress conditions [18,51]. Although the downstream mechanisms vary among tissues, a common feature is the loss of mitochondrial adaptability and stress tolerance. These findings indicate that LONP1 may function as a shared protective factor against chronic organ injury. Importantly, the convergence of mitochondrial dysfunction and fibrotic signaling highlights the potential of LONP1-directed interventions as disease-modifying strategies rather than merely symptomatic treatments [50,62,72].

Although the downstream mechanisms vary among tissues, a common feature is the loss of mitochondrial adaptability and stress tolerance. These findings indicate that LONP1 may function as a shared protective factor against chronic organ injury. Importantly, the convergence of mitochondrial dysfunction and tissue fibrosis highlights the potential of LONP1-directed interventions as disease-modifying strategies rather than merely symptomatic treatments [60,61,71,72].

## 8. Therapeutic Potential of LONP1

The therapeutic relevance of LONP1 varies substantially across disease contexts. While LONP1 inhibition may be advantageous in cancer, restoration of LONP1 activity appears more beneficial in metabolic, degenerative, and fibrotic disorders. A comparative overview of current evidence, therapeutic rationale, and translational challenges is provided in Table 3.

### 8.1. Why Is LONP1 an Attractive Therapeutic Target?

Several characteristics make LONP1 an attractive therapeutic target. First, LONP1 plays a key role within the mitochondrial proteostasis network and regulates multiple biological processes, including protein quality control, mitochondrial bioenergetics, mitochondrial DNA maintenance, stress adaptation, and metabolic remodeling [26,54]. Given the importance of these processes in mitochondrial function, dysregulation of LONP1 can impair cellular metabolism, increase susceptibility to stress-induced injury, and contribute to the pathogenesis of multiple diseases.

Second, accumulating evidence has implicated LONP1 in a broad spectrum of pathological conditions, including cancer, cardiovascular disease, metabolic disorders, neurodegeneration, chronic kidney disease, pulmonary fibrosis, and liver injury [5,7,19]. The involvement of LONP1 across multiple disease contexts suggests that targeting a single mitochondrial regulator may produce systems-level therapeutic benefits.

Third, LONP1 acts upstream of several pathogenic pathways. Through its effects on mitochondrial proteostasis and metabolic adaptation, LONP1 influences oxidative stress, ferroptosis susceptibility, EMT, inflammatory signaling, and metabolic reprogramming [34,35,43]. Consequently, therapeutic modulation of LONP1 may simultaneously affect multiple disease-driving mechanisms.

### 8.2. Therapeutic Inhibition of LONP1

Cancer represents the most compelling setting for LONP1 inhibition. Many tumors exhibit increased dependence on mitochondrial stress adaptation and metabolic flexibility to survive under hypoxic, nutrient-limited, and treatment-induced stress conditions. LONP1 supports these adaptive processes by maintaining mitochondrial proteostasis and bioenergetic homeostasis, thereby promoting tumor cell survival and disease progression [26,34,41].

Experimental studies have demonstrated that suppression of LONP1 disrupts mitochondrial function, induces proteotoxic stress, alters redox balance, and impairs tumor growth [23]. Moreover, LONP1 inhibition has been associated with increased ferroptotic susceptibility and suppression of epithelial–mesenchymal transition, suggesting that targeting LONP1 may simultaneously affect multiple hallmarks of cancer [35,42,43].

Several pharmacological strategies have emerged. CDDO derivatives have been shown to inhibit LonP1 activity through allosteric interference with ATP binding and hydrolysis [73]. More recently, selective small-molecule inhibitors have been developed using structure-based drug design approaches, providing valuable tools for probing LonP1 biology and therapeutic feasibility [74]. In addition, dual targeting of LONP1 and proteasome pathways has demonstrated promising anti-tumor activity in preclinical models, suggesting that simultaneous disruption of mitochondrial and cytosolic proteostasis networks may represent an effective therapeutic strategy [47].

Despite these encouraging findings, several challenges remain. Because LONP1 is essential for normal mitochondrial function, systemic inhibition may cause off-target toxicity in highly metabolic tissues such as the heart, liver, and nervous system. Furthermore, the degree to which cancer cells are selectively dependent on LONP1 remains incompletely understood. Future studies should, therefore, focus on identifying predictive biomarkers and developing cancer-selective LONP1-targeting approaches.

### 8.3. Therapeutic Restoration of LONP1

In contrast to cancer, many degenerative and metabolic disorders may benefit from restoration rather than inhibition of LONP1 activity. Aging-associated decline in LONP1 function contributes to mitochondrial dysfunction, impaired proteostasis, oxidative stress, and progressive tissue degeneration [7,25]. Experimental evidence indicates that reduced LONP1 activity is associated with neurodegeneration, osteoarthritis, sarcopenia, chronic kidney disease, pulmonary fibrosis, and liver injury, suggesting that preservation of mitochondrial quality control may have broad therapeutic implications [56,57,59,60,61,72].

Potential restorative strategies include gene-based therapies, mitochondrial-targeted delivery systems, and pharmacological interventions that enhance LONP1 expression or activity. Although direct LONP1 activators are not currently available, modulation of upstream stress-adaptive pathways may indirectly restore LONP1-dependent mitochondrial resilience. In addition, advances in mitochondrial drug delivery and precision medicine may facilitate tissue-specific targeting of LONP1 in the future.

However, several important limitations must be considered. Excessive LONP1 activation could theoretically promote maladaptive survival pathways or interfere with normal mitochondrial turnover. Furthermore, the optimal therapeutic window is likely to differ among disease contexts. Therefore, future translational efforts should focus on defining disease-specific roles of LONP1 and determining whether therapeutic benefit is best achieved through inhibition, restoration, or context-dependent modulation.

Overall, current evidence suggests that LONP1 is not merely a biomarker of mitochondrial dysfunction but a mechanistically relevant therapeutic target. The dual nature of LONP1 as a pathogenic factor in cancer and a protective factor in degenerative disorders highlights both the opportunities and challenges associated with LONP1-directed therapies.

## 9. Challenges, Emerging Directions, and Translational Perspectives of LONP1 Targeting

Despite the substantial progress made in elucidating LONP1 biology, several critical challenges to its therapeutic translation remain. One major issue is the context-dependent role of LONP1 across diseases. While LONP1 preserves mitochondrial integrity and cellular survival in aging and metabolic disorders, it simultaneously supports tumor growth, metabolic reprogramming, and therapy resistance in cancer [8,19,23]. This functional duality highlights the need for context-specific and tissue-selective modulation rather than global inhibition or activation.

At the mechanistic level, the substrate selectivity and regulatory logic of LONP1 remain incompletely defined. Recent structural studies using cryo-electron microscopy have revealed dynamic ATP-driven conformational cycling and substrate-threading mechanisms [6,12], yet the determinants that govern the balance between proteolysis and chaperone-like activity remain unclear. Emerging proteomic analyses further suggest that LONP1 selectively regulates metabolic enzymes and stress-responsive proteins in a context-dependent manner [36,68]. In addition, growing evidence indicates that LONP1 operates within a broader mitochondrial proteostasis network, interacting with CLPP, mtHSP70, and mitophagy pathways to coordinate adaptive responses [2,41]. Another emerging frontier is the role of LONP1 in inter-organelle communication. Recent studies demonstrate that LONP1 contributes to MAM integrity and coordinates the UPRmt with the UPRER, thereby linking mitochondrial dysfunction to global cellular stress adaptation [18,69]. These findings suggest that LONP1 functions not only as a mitochondrial protease but also as a regulator of cellular stress integration, although the precise signaling circuits involved have yet to be fully elucidated. From a translational perspective, the strategy of pharmacologically targeting LONP1 is still in an early stage of development but is rapidly evolving. Small-molecule modulators that inhibit or finetune LONP1 activity have shown potential in disrupting mitochondrial proteostasis and inducing cancer cell vulnerability [8,75]. Combination strategies, such as co-targeting LONP1 and the proteasome or metabolic pathways, have demonstrated enhanced anti-tumor efficacy in preclinical models [47]. Conversely, in aging and degenerative diseases, the restoration or enhancement of LONP1 function may improve mitochondrial resilience, reduce proteotoxic stress, and restore metabolic homeostasis [3,62]. This bidirectional therapeutic paradigm underscores the importance of precision mitochondrial medicine.

Looking forward, the integration of multi-omics approaches, including mitochondrial proteomics, metabolomics, and single-cell transcriptomics, will be essential to define LONP1-dependent regulatory networks across tissues and disease contexts. In parallel, the development of tissue-specific delivery systems and condition-dependent modulators may enable selective targeting of LONP1 while minimizing systemic toxicity. Ultimately, a deeper understanding of LONP1-centered regulatory networks will be critical for translating mitochondrial proteostasis biology into clinically actionable strategies.

## 10. Discussion and Future Perspectives

Taken together, the available evidence indicates that LONP1 influences multiple aspects of mitochondrial function, including protein quality control, metabolism, and cellular stress responses, with important implications for human disease. Beyond its canonical role in mitochondrial protein degradation, LONP1 influences diverse aspects of mitochondrial function, including protein quality control, energy metabolism, mitochondrial DNA maintenance, and cellular stress responses. This interpretation is supported by accumulating evidence indicating that mitochondrial proteases influence diverse biological processes, including protein quality control, metabolic regulation, and cellular stress responses [17,76,77].

Within this framework, LONP1 can be viewed as a proteostasis-buffering hub that defines the threshold of mitochondrial stress tolerance [18]. Under homeostatic conditions, LONP1 maintains proteome integrity and supports efficient OXPHOS, thereby enabling metabolic flexibility [20]. However, during aging or chronic stress, the progressive impairment of LONP1 disrupts this buffering capacity, leading to misfolded protein accumulation, respiratory chain complex destabilization, and amplified mtROS generation [21,23,68,78]. These alterations establish a self-reinforcing cycle of proteotoxic and redox stress, driving mitochondrial dysfunction and cellular decline. This model is supported by studies demonstrating that disruption of mitochondrial quality control is associated with aging-related functional decline and tissue degeneration [77,79].

Importantly, LONP1 operates within an interconnected mitochondrial proteostasis network rather than as an isolated factor, coordinating PQC with mtDNA maintenance, mitochondrial transcription, mitoribosomal translation, and OXPHOS complex assembly [80]. Functional crosstalk with mitochondrial chaperones, mitophagy pathways, and stress response programs, such as the UPRmt, establishes a multilayered adaptive circuit that coordinates protein folding, proteolysis, and organelle turnover. This integrated network enables mitochondria to dynamically buffer proteotoxic stress, modulate ROS signaling, and finetune mitophagic flux to preserve organelle integrity and cellular fitness [17,19,81,82,83,84]. Perturbation of this network may shift the balance from adaptive remodeling toward maladaptive stress responses, emphasizing that mitochondrial dysfunction arises from network collapse rather than from single-node failure.

LONP1 enhances cellular fitness by buffering mitochondrial proteotoxic stress and sustaining metabolic plasticity through the regulation of mitochondrial PQC and bioenergetic remodeling. These functions are consistent with the framework of non-oncogene addiction, whereby cancer cells become highly dependent on stress response systems, including mitochondrial proteostasis and UPRmt pathways, to tolerate proteotoxic, oxidative, and metabolic stress and maintain survival [5,23,81,85]. Contrastingly, in aging and degenerative disorders, LONP1 insufficiency drives mitochondrial decline by disrupting mitochondrial proteostasis, resulting in the accumulation of misfolded proteins, impaired respiratory capacity, and progressive bioenergetic failure. These defects ultimately translate into tissue dysfunction across multiple organ systems, including metabolic, musculoskeletal, and neurodegenerative contexts [18,22,25,57,85]. This functional duality highlights the importance of precision mitochondrial medicine, in which LONP1 activity must be selectively modulated according to the disease context, metabolic state, and cellular stress burden.

Despite these advances, several critical gaps remain. The molecular determinants governing LONP1 substrate specificity and the regulatory switch between its proteolytic and chaperone-like activities are still incompletely defined, despite recent structural and biochemical insights into AAA^+^ proteases and mitochondrial quality control systems [7,11,12]. In addition, the mechanisms through which LONP1 coordinates intra-mitochondrial proteostasis with extra-mitochondrial signaling, particularly at MAMs, remain poorly understood, although emerging evidence suggests a role for mitochondrial proteases in inter-organelle communication and stress signaling [22,69,86]. Furthermore, the lack of selective and clinically viable LONP1 modulators continues to impede translational progress, as only a limited number of small-molecule inhibitors have been identified to date, many of which exhibit suboptimal specificity and pharmacological properties. Current efforts remain largely confined to early-stage preclinical development, with candidate compounds, such as CDDO derivatives and emerging structure-guided inhibitors, yet to advance beyond experimental validation [7,8,47,73,74,75,87]. Addressing these challenges will require the integration of structural biology, spatial proteomics, and systems-level approaches to delineate LONP1-centered regulatory networks across tissues and disease states [5,19]. Looking ahead, advances in multi-omics technologies and single-cell analyses are expected to refine our understanding of LONP1 as a context-dependent regulator of mitochondrial adaptation, enabling the resolution of cell type-specific and stress-dependent mitochondrial states [88,89,90]. Coupled with the development of mitochondria-targeted delivery systems and condition-specific modulators of mitochondrial proteostasis, these approaches may enable selective and spatiotemporally controlled manipulation of LONP1 activity [10,48,91], particularly given the emerging integration of mitochondrial quality control with broader cellular stress signaling networks and inter-organelle communication pathways. Ultimately, positioning LONP1 within a systems-level framework of mitochondrial resilience not only advances our understanding of mitochondrial biology but also provides a conceptual foundation for developing novel therapeutic strategies aimed at restoring cellular homeostasis across a wide spectrum of human diseases [76,92].

Collectively, the evidence reviewed herein supports a model in which LONP1 functions as a central regulator of mitochondrial adaptation by integrating proteostasis, metabolic regulation, mitochondrial genome maintenance, and stress-responsive signaling. Rather than acting solely as a mitochondrial quality control protease, LONP1 coordinates multiple interconnected pathways that collectively determine mitochondrial resilience under physiological and pathological conditions. The context-dependent consequences of LONP1 dysregulation across cancer, metabolic disorders, aging, and degenerative diseases further highlight its importance as a mitochondrial adaptive hub. Future studies should focus on defining tissue-specific regulatory mechanisms, identifying disease-relevant substrates, and developing selective strategies to modulate LONP1 activity. Such advances may facilitate the translation of LONP1-targeted interventions for the treatment of mitochondrial dysfunction-associated diseases.

## Figures and Tables

**Table 1:** LONP1 in cancer: mechanisms and evidence.

Major Category	Subcategory	Molecular Mechanism	Functional Outcome	Cancer Context	References
Tumor-Promoting Mechanisms	Metabolic reprogramming	MPC1 degradation; mitochondrial enzyme remodeling	Glycolytic shift; metabolic flexibility	Prostate cancer; metabolic diseases	[22,34]
	Redox adaptation	ROS modulation; ETC regulation; signaling activation	MAPK activation; EMT; survival	Multiple cancers	[23,40]
	Proteostasis buffering	Degradation of misfolded proteins; cooperation with CLPP	Anti-proteotoxic stress; mitochondrial integrity	Broad cancer types	[23,41]
	Ferroptosis regulation	ACO2 degradation; GPX4/Nrf2 modulation	Context-dependent ferroptosis sensitivity	HCC; pancreatic cancer	[35,42]
	EMT and tumor progression	JNK signaling; ROS-mediated pathways	Migration; invasion; metastasis	Pancreatic; colorectal; cervical	[43,44,45]
Adaptive Cancer Phenotypes	Therapeutic resistance	Proteasome compensation; dual inhibition sensitivity	Drug resistance; enhanced survival	Multiple myeloma; astrocytoma	[46,47]

ACO2, aconitase 2; CLPP, caseinolytic mitochondrial matrix peptidase proteolytic subunit; EMT, epithelial–mesenchymal transition; ETC, electron transport chain; GPX4, glutathione peroxidase 4; HCC, hepatocellular carcinoma; JNK, c-Jun N-terminal kinase; LONP1, mitochondrial Lon peptidase 1; MAPK, mitogen-activated protein kinase; MPC1, mitochondrial pyruvate carrier 1; Nrf2, nuclear factor erythroid 2-related factor 2; ROS, reactive oxygen species.

**Table 2:** Role of LONP1 in metabolic and systemic diseases.

Disease Context	Molecular Mechanism	Functional Consequence	Biological/Clinical Outcome	References
Type 2 diabetes (β-cells)	LONP1 deficiency impairs mitochondrial proteostasis and protein folding	Mitochondrial dysfunction; reduced OXPHOS	β-cell apoptosis; impaired insulin secretion; glucose intolerance	[22,49]
Cardiac development	LONP1 maintains mitochondrial proteostasis during metabolic transition	Supports shift to OXPHOS	Proper cardiac maturation and function	[9]
Cardiac stress	LONP1 regulates mitochondrial stress responses and redox balance	Maintains ETC function; limits oxidative damage	Protection against cardiac dysfunction and ischemic injury	[18,51]
MASH-associated liver fibrosis	LONP1 regulates DHODH-dependent metabolism	Prevents orotic acid accumulation	Reduced hepatic stellate cell activation and fibrosis	[52]
Skeletal muscle	LONP1 deficiency induces mitochondrial stress signaling	Activates systemic adaptive pathways	Improved metabolic resilience under stress	[53]
ER–mitochondria crosstalk	LONP1 coordinates UPRmt and UPRER signaling	Maintains inter-organelle proteostasis	Preserves cellular homeostasis in high-energy tissues	[54]
General metabolic homeostasis	LONP1 integrates proteostasis with metabolic regulation	Maintains mitochondrial function under stress	Prevents metabolic and organ dysfunction	[19]

DHODH, dihydroorotate dehydrogenase; ER, endoplasmic reticulum; ETC, electron transport chain; LONP1, mitochondrial Lon peptidase 1; MASH, metabolic dysfunction-associated steatohepatitis; OXPHOS, oxidative phosphorylation; UPRER, endoplasmic reticulum unfolded protein response; UPRmt, mitochondrial unfolded protein response.

**Table 3:** Therapeutic relevance of LONP1 across different pathological contexts.

Pathological Context	Role of LONP1	Key Mechanism	Therapeutic Strategy	Potential Benefit	Evidence	Key Limitation	References
Type 2 Diabetes	Protective	Maintains β-cell mitochondrial quality control	Restore LONP1 activity	Improved β-cell survival and insulin secretion	Experimental and animal studies	No validated activators	[3,49]
Cardiovascular Disease	Protective	Preserves mitochondrial homeostasis	Enhance LONP1 function	Reduced oxidative stress and cardiac injury	Animal studies	Limited translational evidence	[18,51,54]
Cancer	Pathogenic (upregulated)	mitochondrial function, metabolic adaptation, and therapy resistance	Inhibit LONP1	Reduced tumor growth and improved treatment response	Cell and animal studies	No clinical trials	[26,34,35,40,41]
Osteoarthritis and Musculoskeletal Aging	Protective	Maintains mitochondrial integrity	Restore LONP1 activity	Delayed tissue degeneration	Experimental studies	No therapeutic validation	[52,57]
Neurodegenerative Disorders	Protective	Prevents mitochondrial protein damage	Restore LONP1 function	Neuroprotection and cognitive preservation	Experimental and animal studies	No disease-specific therapies	[56,63,64]
Chronic Kidney Disease/Renal Fibrosis	Protective	Limits mitochondrial dysfunction and fibrosis	Restore LONP1 function	Reduced fibrosis and renal damage	Animal studies	Long-term efficacy unknown	[60,71]
Liver Injury and Metabolic Liver Disease	Protective	Supports mitochondrial metabolism	Enhance LONP1 activity	Reduced liver injury	Experimental and animal studies	Limited clinical evidence	[52,72]
Pulmonary Fibrosis	Protective	Prevents mitochondrial dysfunction	Restore LONP1 activity	Slower fibrotic progression	Experimental studies	Early-stage evidence	[61]
Aging	Protective	Maintains mitochondrial proteostasis and bioenergetics	Preserve or enhance LONP1 activity	Improved mitochondrial resilience	Experimental studies	No clinical evidence	[7,25,62]

## Data Availability

No new data were created or analyzed in this study. Data sharing is not applicable to this article.

## Abbreviations

The following abbreviations are used in this manuscript:

AAA^+^	ATPases associated with diverse cellular activities
ATF3	Activating transcription factor 3
CLPP	Caseinolytic mitochondrial matrix peptidase proteolytic subunit
DHODH	Dihydroorotate dehydrogenase
EMT	Epithelial–mesenchymal transition
ER	Endoplasmic reticulum
ETC	Electron transport chain
LONP1	Lon peptidase 1, mitochondrial
MAPK	Mitogen-activated protein kinase
MAM	Mitochondria-associated membrane
MPC1	Mitochondrial pyruvate carrier 1
mtDNA	Mitochondrial DNA
mtHSP70	Mitochondrial heat shock protein 70
mtROS	Mitochondrial reactive oxygen species
OXPHOS	Oxidative phosphorylation
PQC	Protein quality control
ROS	Reactive oxygen species
TFAM	Mitochondrial transcription factor A
UPRER	Endoplasmic reticulum unfolded protein response
UPRmt	Mitochondrial unfolded protein response
